# In-Field *Diadegma insulare* (Cresson) (Hymenoptera: Ichneumonidae) Parasitism Rates of *Plutella xylostella* (L.) (Lepidoptera: Plutellidae) in Virginia Cole Crops

**DOI:** 10.3390/insects17030268

**Published:** 2026-03-03

**Authors:** Taylore A. Tomlinson, Alejandro I. Del Pozo-Valdivia, Thomas P. Kuhar

**Affiliations:** 1 Department of Entomology, Virginia Tech, Blacksburg, VA 24061, USA; 2 Department of Entomology, Virgina Tech Hampton Roads AREC, Virgina Beach, VA 23455, USA; adelpozo@vt.edu

**Keywords:** biological control, diamondback moth, parasitoids, natural enemies, in-field surveys

## Abstract

The diamondback moth, *Plutella xylostella* (L.), is a significant pest of brassica crops that is found across the globe. Due to the development of insecticide resistance, control tactics have shifted focus towards integrating pest management techniques such as biological control. *Diadegma insulare* (C.), *Oomyzus sokolowskii* (K.), and *Microplites plutellae* (M.) are parasitoids of *P. xylostella* found in the Eastern United States. From 2022 to 2025, we surveyed *P. xylostella* larvae and pupae in locations across Virginia to assess the current rates of parasitism in brassica fields. Specimens were brought to the laboratory and reared to assess parasitoid emergence rates. Only *D. insulare* specimens were found during the study. Adult *P. xylostella*, larvae and pupae, adult *D. insulare*, *D. insulare* pupae, unknown parasitoids, and unknown deaths were recorded and used to calculate the rates of parasitism at each location. We concluded that the parasitism rate varied by location and year, which was expected due to regional conditions and seasonality. Rates averaged between 30.1 and 65% by year, with the lowest individual rate being 15% in 2025 and the highest at 100% in 2022. This suggests that *D. insulare* is actively present in Virginia and could be a successful biological control agent when paired with other integrated pest management techniques to reduce *P. xylostella* populations.

## Simple Summary

The diamondback moth, *Plutella xylostella* (L.), is a pest of brassica crops that is found around the world and is challenging to manage due to insecticide resistance. To manage this pest, alternative integrated pest management techniques are being explored. Biological control of the diamondback moth by parasitoid wasps, such as *Diadegma insulare*, *Oomyzus sokolowskii*, and *Microplites plutellae*, has been previously studied in the literature. However, there are no current records of parasitism rate activity on *P. xylostella* in Virginia in the past 15 years. This information is crucial to determine the role of biological control agents in managing *P. xylostella* and whether it could potentially be used as a control strategy. We sampled locations across Virginia from 2022 to 2025 to survey the current parasitism rates of *P. xylostella* on brassica farms. The percentage rates of parasitism averaged between 30.1 and 65% each year. The lowest rate of parasitism was 15% in 2025, with the highest at 100% in 2022. Overall, our findings suggest that *D. insulare* is currently playing a role in *P. xylostella* management in Virginia and has the potential to be successful as a biological control agent when paired with other management strategies. No other parasitoids were found during the study.

## 1. Introduction

The diamondback moth, *Plutella xylostella* (L.) (Lepidoptera: Plutellidae), is a significant pest of brassica (*Brassica* spp.) crops in most parts of the world [1], including all vegetable-growing regions of the United States [2]. *Plutella xylostella* can be particularly difficult to manage due to its rapid ability to develop insecticide resistance [3]. This pest is already resistant to over 100 insecticides used to manage lepidopteran pests in brassica systems [4,5]. The current challenge of insecticide resistance has shifted the focus to developing integrated pest management (IPM) practices for the management of *P. xylostella*, including the augmentation and conservation of biological control [1,2,6,7]. Biological control can be a complementary control tactic to help reduce resistant lepidopteran pest populations that remain after insecticide applications.

Hymenoptera parasitoids are the most extensively studied and can be impactful in controlling *P. xylostella* populations, with numerous species previously reported [1,6,8]. Twenty-two species of parasitoids of *P. xylostella* have been described around the globe [9]. Three commonly reported larval parasitoids of *P. xylostella* in North America are *Diadegma insulare* (Cresson) (Hymenoptera: Ichneumonidae), *Microplites plutellae* (Muesebeck) (Hymenoptera: Braconidae), and *Oomyzus sokolowskii* (Kurdjumov) (Hymenoptera: Eulophidae) [10,11,12,13,14,15,16,17,18]. These three species have also been reported in Virginia, but very little is known about their current parasitism rates. *Diadegma insulare* is likely the most common *P. xylostella* parasitoid found in fields in the Eastern United States, but current surveys are lacking [18]. Adult *D. insulare* and *M. plutellae* are solitary wasps and host-specific, unlike other species, such as *O. sokolowskii*, which is gregarious with a large host range [14,18,19,20]. Parasitism by *D. insulare* occurs when the adult female parasitoid wasp inserts her ovipositor into the host’s larval body and deposits eggs. Once the host pupates, the parasitoid eggs will hatch and the larva will exit the host body to pupate, resulting in host death. The parasitoid wasp larvae will use the pupal casings of its host for protection [21].

Parasitism rate surveys could be used to determine the species complex, incidence, and relative importance of parasitoids with regard to the biological control of a pest organism. Determining the rates of parasitism of target hosts can be difficult due to the nature of endoparasitoids developing within the larval host body. Additionally, it would be difficult to determine the abundance of parasitoids in each region due to their small size and vulnerability to detrimental conditions, such as weather conditions or insecticide spraying [22,23]. Parasitism rate surveys are used to determine the parasitism of a subsample of a target pest, such as *P. xylostella*, *Helicoverpa zea* (Boddie) (Lepidoptera: Noctuidae), or *Spodoptera frugiperda* (Smith) (Lepidoptera: Noctuidae) [24,25]. These surveys can be conducted in three different ways: (1) rearing the parasitoid from a host, (2) dissecting field-collected host larvae to determine parasitoid presence [26], or (3) using PCR-based identification methods [27].

*Diadegma insulare* has been reported across the United States through parasitism surveys of *P. xylostella* populations in brassica systems. In the late 1980s and early 1990s, the parasitism rates of *D. insulare* were found to be as high as 88% in East Lansing, Michigan, during peak growing season [28,29]. In Santa Cruz, California, the parasitism rates of *D. insulare* were as high as 70–100% [30]. However, in the Eastern United States, low to moderate rates of parasitism have been recorded when surveyed in Gainesville, Florida (3–72%) and Geneva, New York (15–46%) [16,31]. All of these studies were conducted in the summer, during warm weather and at peak population densities. However, recent studies have not been conducted to determine up-to-date parasitism rates of *P. xylostella* in Virginia or other mid-Atlantic states.

Research on the topic of parasitism is crucial to determine whether this form of biological control contributes to managing *P. xylostella* populations alongside proper insecticide active ingredient selection and timing in Virginia during the growing season. The use of biological control alongside other control practices can help growers to manage *P. xylostella* populations and reduce the risk of further insecticide resistance development. The goal of this study was to determine the parasitism rates of *D. insulare* and other potential parasitoid species on field-collected *P. xylostella*, providing information about the significance of biological control of *P. xylostella* in brassica systems in Virginia.

## 2. Materials and Methods

**Figure 1:**
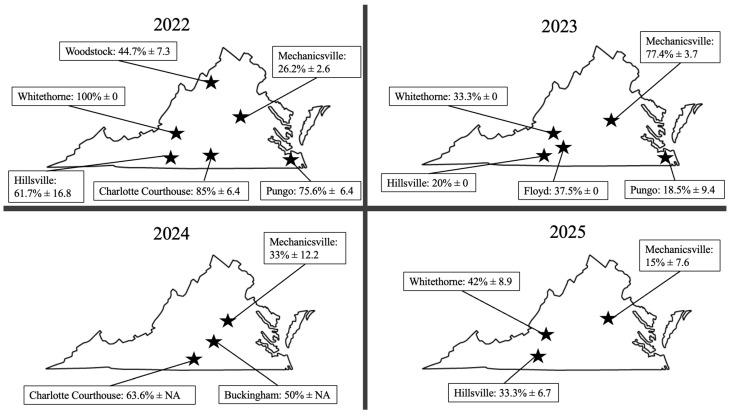
Field site map with average percent parasitism rate ± standard error of field-collected *P. xylostella* specimens for *D. insulare* sampled in Virginia by year.

Parasitism surveys were focused on the collection of *P. xylostella* larvae and pupae from conventional commercial and organic brassica fields. Eight field sites were selected based on the known presence of *P. xylostella* in different regions of Virginia (2022–2025). Sites included Woodstock, Pungo, Hillsville, Whitethorne, Mechanicsville, Floyd, Charlotte Court House, and Buckingham (Figure 1). Field sites were commercial, conventional brassica fields, consisting of green cabbage, collards, and broccoli, except for Floyd, which was a small-production organic farm (Table 1). Field sites were managed at the discretion of our grower collaborators. With the exception of the organic field site in Floyd, commercial practices also included the spraying of pesticides in these fields. At each location, the number of *P. xylostella* specimens collected varied due to density. A minimum of 10 larvae or pupae were collected per field site, since collections happened once per field site. Collected specimens were brought back to the laboratory in Blacksburg, Virginia and separated into Petri dishes in groups of five specimens with a fresh untreated cabbage leaf or with filter paper for pupae [Thermo Fisher Scientific, Waltham, MA, USA]. Petri dishes were checked for emergence every 48 h for 7 days to allow enough time for specimens to eclose. At each check, the numbers of adult *P. xylostella*, *P. xylostella* larvae, *P. xylostella* pupae, live *D. insulare* adults, *D. insulare* pupae, other parasitoids, and unknown deaths of specimens were recorded. Parasitoids were identified through keys provided by the American Entomological Institute (2015).

Percent parasitism rates were calculated using the following formula for each location:

$$\mathrm{Parasitism}\;\mathrm{rate}\;(\%)\;=\;100\times (\frac{\mathrm{Number}\;\mathrm{parasitoids}\;\mathrm{emerged}}{\mathrm{Number}\;\mathrm{parasitoids}\;\mathrm{emerged}\;+\;P.\;\mathrm{xylostella}\;\mathrm{pupae}})$$

Percentage rates were averaged across all dishes from a single location. Percentage means and standard errors were calculated using the package ‘dplyr’ in R (R Studio Team 2023, version 2023.12.1+402). These means were not modeled or compared statistically between locations due to variability in regional conditions. Additionally, each field site was managed differently, with varying soil types and cultural practices. Unknown death rates were determined when *P. xylostella* larvae were moribund with no signs of parasitism and were recorded.

## 3. Results

In 2022, across all locations, 434 *P. xylostella* larvae were collected, with 178 reared *D. insulare.* The number of unknown dead specimens recorded was 81. This resulted in the overall mean percentage of parasitism being 65% in 2022. The mean *P. xylostella* parasitism rates for *D. insulare* varied across the sampling locations. Whitethorne displayed the highest rate of parasitism (100%), while Mechanicsville displayed the lowest rate (26.2%). Moderate to high levels of parasitism were observed in Charlotte Courthouse (85.0%), Pungo (75.6%), Hillsville (61.7%), and Woodstock (44.7%) (Figure 1).

In 2023, a total of 258 *P. xylostella* specimens were collected, with 29 unknown deaths and 89 *D. insulare* emerged. The mean parasitism rates were lower in some of the sampling locations compared to the previous year. The collected specimens of *P. xylostella* showed the following mean rates of parasitism: Mechanicsville 77.4%, Floyd 37.5%, Whitethorne 33.3%, Hillsville 20.0%, and Pungo 18.5% (Figure 1). Overall, the average parasitism rate in 2023 was 37.3%. In 2024, only three locations in Virginia were sampled, with a total of 417 *P. xylostella* collected and 119 *D. insulare* emerged, and 51 unknown deaths were recorded. The overall average parasitism rate for the 2024 surveys was 49.0%. The average parasitism rates varied, with the highest in Charotte Courthouse (63.6%), followed by Buckingham (50.0%) and Mechanicsville (33.0%) (Figure 1).

Similarly, in 2025, only three locations were sampled in Virginia due to low larval presence and reduced pest pressure. In total, 62 *P. xylostella* specimens were collected, with 21 *D. insulare* emerged specimens. Whitethorne showed the highest parasitism (42.0%), followed by Hillsville (33.3%) and Mechanicsville (15.0%) (Figure 1). The average overall parasitism rate in 2025 was 30.1%. In 2025, 18 unknown deaths were recorded. Across all field sites and all years, a total of 1171 *P. xylostella* and 407 *D. insulare* were collected over four years for this study. No *O. sokolowskii* or other parasitoids were recovered from any of our samples across all field sites and all years.

## 4. Discussion

*Plutella xylostella* is a challenging pest to manage in brassica systems due to insecticide resistance. Our results from in-field parasitism rate surveys showed relatively high parasitism levels across Virginia. The highest rates were recorded in 2022 in Whitethorne at 100.0% and in Charlotte Court House at 85.0%. These rates were recorded from locations under conventional insecticide application regimens, which could explain the potential resiliency of *D. insulare* to still parasitize *P. xylostella* under adverse conditions. Additionally, the recorded parasitism rates never fell below 15.0%, showing that *D. insulare* is an active biological control agent in Virginia. As an important IPM practice, it is crucial to determine whether conventional or organic insecticidal applications have a significant effect on the ability of parasitoids to find and attack their hosts.

Effective IPM requires the incorporation and fostering of natural enemy populations by reducing the number of spray applications and applying insecticides with limited non-target effects. Furthermore, conducting insecticide applications when natural enemy populations are low or not present would be beneficial for the implementation of an IPM program that aims to promote conservational biological control. Given the frequent use of insecticides in brassica systems, it is important to understand the effects of the applied insecticides on parasitoids as part of a sound pest management program [18]. Organic insecticides may pose a lower risk to *D. insulare*. In early experiments, *D. insulare* was shown to be able to effectively parasitize *P. xylostella* and was unaffected by applications of spinosad [32]. Furthermore, Xu et al. (2004) tested the effects of azadiractin-based insecticides on *D. insulare* in treated Petri dishes, which resulted in 0.0–10.4% mortality [33]. Specimens of *D. insulare* that survived the organic treatments were able to parasitize 50.8–67.6% of collected *P. xylostella* larvae [33]. However, azadiractin-based insecticides are not commonly used for *P. xylostella* management. Cordero et al. (2007) studied disks under laboratory conditions in which cabbage leaf was treated with broad-spectrum insecticides such as esfenvalerate (pyrethroid), methomyl (carbamate), or acephate (organophosphate), which resulted in 100.0% mortality among adults of *D. insulare* and *O. sokolowskii* at field rate concentrations after 72 h [18]. Additionally, more IPM-compatible insecticides, such as emamectin benzoate, spinosad, and indoxacarb, also resulted in 100% mortality among both parasitoid species after 72 h [18]. In contrast to the studies by Hill and Foster (2000) [32], Cordero et al. (2007) [18] found complete mortality of *O. sokolowskii* and *D. insulare* when exposed to field rate concentrations of spinosad, an organic insecticide, after 72 h. Cordero et al. (2007) used a much higher rate of spinosad (73.2 mg) and monitored insects for up to 72 h, while Hill and Foster (2000) used a lower rate of spinosad (0.31 mg) and monitored for 24 h [18,32].

There are several potential factors influencing the variability in the parasitism rates documented across the sampling locations in this study. Each location may have different growing seasons, host plants, pest densities, weather and climate conditions, soil types, commercial standard practices, spray application types and timing, and other environmental factors that could have affected the abundance of these parasitoids [34]. Sampling for *P. xylostella* specimens also varied regarding the time of the year, where larvae were mostly collected at the peak of the growing season at each location. Additionally, a few locations showed a drop in parasitism rates from the initial sampling year. In 2022, Whitethorne showed 100% parasitism but this dropped significantly to 33% (2023) and 42% (2024) in the following years. In 2025, only 62 *P. xylostella* specimens were collected from three different locations. These changes in parasitism rates and *P. xylostella* abundance could have been the result of intensive spray regimens or harsh weather conditions, such as high rainfall.

The question remains as to why *P. xylostella* is still a significant problem in brassica systems if these parasitoids and heavy spray applications are present. It can be difficult to rely on successful biological control alone. Biological control agents, such as *D. insulare*, require proper timing to parasitize their hosts. *Diadegma insulare* has a preference for parasitizing the fourth instar of *P. xylostella* [17]. If synchrony in generations between the parasitoid and host is not present, it can be difficult to achieve high rates of parasitism [35,36,37]. Additionally, if a field site has been heavily sprayed with insecticides, *P. xylostella* may show some resistance, while biological control agents like *D. insulare* and *O. sokolowskii* have shown high susceptibility to many conventional active ingredients [5,18]. It is important to use IPM practices to help manage *P. xylostella*, reducing the risks of resistance and conserving biological control agents.

Throughout this study, unknown deaths of collected *P. xylostella* specimens were recorded. Across four years of sampling and rearing *P. xylostella*, a range of 18–81 unknown deaths occurred. This could have been the result of the cross-contamination of insecticides on the leaf discs used to rear *P. xylostella* specimens to adulthood in the laboratory. Leaves were obtained from green cabbage heads bought from the organic section of the local grocery store and washed thoroughly. Collected specimens were stored in a cooler during transport from the sampling location to the laboratory; this often required four or more hours of driving, which could have cause stress in the larvae. Furthermore, the sampled field locations could have been recently treated with insecticides, which may have been present on *P. xylostella* specimens when they were collected.

It is important to note that *D. insulare* was the only parasitoid found in all of the samples across four years in Virginia. *Oomyzus sokolowskii* and *M. plutellae* have also been previously recorded in the Eastern United States but were not found during this survey [11,15]. A possible limitation of the study methodology is that *O. sokolowskii* and *M. plutellae* can take up to 3–4 weeks to eclose from pupae [20]. Furthermore, there can be differences in population peaks for *O. sokolowskii* and *M. plutellae* depending on regionality. The results of this survey showed that 407 *D. insulare* emerged from 1171 *P. xylostella* specimens collected, demonstrating a high abundance of *D. insulare* populations across Virginia. A total of 34% of all *P. xylostella* collected across the four years and collection sites were parasitized by *D. insulare*. These results demonstrate the importance of *D. insulare* as a biological control agent of *P. xylostella*, as well as the value of integrated pest management tactics that conserve this biological control agent.

The conservation of biological control agents such as *D. insulare* can be achieved through well-planned pest management practices and by minimizing significant non-target effects. The timing and number of insecticidal applications can have an effect on natural enemy populations. If broad-spectrum insecticides are sprayed during peak hours of natural enemy activity, there is a chance that these populations could be harmed. Additionally, a high rate of application could reduce natural enemy populations after multiple insecticides are sprayed. It is important to determine the insecticides with the greatest potential to harm biological control agents, considering previous information from bioassays and insecticide treatment experiments [18,38,39,40,41].

*Diadegma insulare* also may have the potential to serve as an augmentative biological control agent, but further studies are needed to understand the impacts of releasing large numbers of *D. insulare* into the field. Augmentative biological control can be costly, and success can be strongly influenced by economical barriers and non-target risks, such as release rates or timing, intensive insecticidal applications, regulation policies, mass rearing challenges, and weather conditions [42].

## Figures and Tables

**Table 1:** Field site variables by location and sample date. Each location was maintained at the grower’s discretion.

Location	Sample Date	Farm Type	Crop Cultivar	Row Spacing	Field Size	Specimens Collected
Mechanicsville	06/09/2022 25/04/2023 06/06/2024 09/06/2025	Conventional	Broccoli: Eastern Crown, Commonwealth Cabbage: Blue Vantage, Gregorian	Single plant rows 1 m apart, 0.3 m plant spacing	14.16 ha	519
Hillsville	27/07/2022 07/08/2023 16/10/2025	Conventional	Cabbage: Savoy, Brennan, Bruno, Ramada	1.8 m beds of 3 seeding rows, 0.5 m plant spacing	4.04–8 ha	65
Charlotte Courthouse	07/09/2022 25/09/2024	Conventional	Cabbage: Savoy, Brennan, Bruno, Ramada	Single plant rows 1 m apart, 0.3 m plant spacing	6.07 ha	95
Pungo	21/06/2022 24/04/2023	Conventional	Cabbage: Ramada, Bruno, Blue Vantage Broccoli: Eastern Crown and Commonwealth	Single plant rows 1 m apart, 0.3 m plant spacing	2.02 ha	377
Whitethorne	02/09/2022 07/07/2023	Conventional	Cabbage: Bronco	6 m beds of 1 plant row, 0.3 m plant spacing	0.4 ha	87
Woodstock	29/06/2022	Conventional	Cabbage: Ramada, Bronco, Savoy, Red Cabbage	Single plant rows 1 m apart, 0.3 m plant spacing	4.04 ha	52
Floyd	15/06/2023	Organic	Cabbage: Red Cabbage, Savoy, Late Flat Dutch, Brunswick	Single plant rows 1 m apart, 0.3 m plant spacing	0.81 ha	13
Buckingham	16/05/2024	Conventional	Cabbage: Savoy, Brennan, Bruno	Single plant rows 1 m apart, 0.3 m plant spacing	2.02 ha	11

## Data Availability

The raw data supporting the conclusions of this article will be made available by the authors on request.
